# Complexities in consultations in case of euthanasia or physician-assisted suicide: a survey among SCEN physicians

**DOI:** 10.1186/s12875-019-1063-z

**Published:** 2020-01-09

**Authors:** Tessa D. Bergman, H. Roeline W. Pasman, Bregje D. Onwuteaka-Philipsen

**Affiliations:** Department of Public and Occupational Health, Amsterdam Public Health Research Institute, Amsterdam UMC, Vrije Universiteit Amsterdam, P.O. Box 7057, 1007 MB Amsterdam, The Netherlands

**Keywords:** Euthanasia, Assisted suicide, SCEN physician, Euthanasia consultation, Euthanasia law

## Abstract

**Background:**

In the Netherlands, euthanasia or physician-assisted suicide (EAS) is allowed if due care criteria are met. One criterion is consultation of a second independent physician, often SCEN physicians. The public debate about EAS focuses on patients with psychiatric disorders, dementia, and tired of living, as complex cases. What complexities SCEN physicians perceive during consultation is unknown. This study aims to assess the frequency of EAS consultations that are perceived difficult by SCEN physicians, to explore what complexities are perceived by SCEN physicians during consultation, and to assess what characteristics are associated with difficult consultations.

**Methods:**

Data from 2015 to 2017 from an annual cross-sectional survey among SCEN physicians was used. In 2015, the survey focused on the most difficult consultation that year and in 2016/2017 on the most recent consultation. Frequencies of coded answers to an open-ended question were done to explore what complexities SCEN physicians perceived during their most difficult consultation. Univariable and multivariable logistic regression analyses were used to assess what characteristics were associated with difficult consultations.

**Results:**

21.6% of cases consulted by SCEN physicians is perceived difficult. Complexities that SCEN physicians perceive were mainly in contact with patients (79.7%) and in the assessment of due care criteria (41.0%). Characteristics that were associated with a higher likelihood of a consultation being difficult are the attending physician being less certain to perform the EAS, patients staying in the hospital, main diagnosis heart failure/CVA, and accumulation of age-related health problems/psychiatry/dementia, and the presence of a psychiatric disorder, or psychosocial or existential problems besides the main diagnosis. Characteristics that were associated with a lower likelihood of a consultation being difficult are high patient’s age and physical suffering as reason to request EAS.

**Conclusion:**

Complexities perceived by SCEN physicians in EAS consultations are not limited to the ‘complex’ cases present in the current public debate about EAS, e.g. patients with psychiatric disorders, dementia, and tired of living. Attention for these complexities in intervision could indicate if there is a need among SCEN physicians to enhance knowledge and skills in training and to receive specific support in intervision on these complexities.

## Background

In 2015, 4.6% of deaths in the Netherlands was due to euthanasia or physician-assisted suicide (EAS) [1]. EAS is regulated under the Dutch Euthanasia Act (2002): a physician will not be prosecuted for performing EAS if the due care criteria are met and he/she reports the EAS to the Regional Euthanasia Review Committee [2]. The due care criteria are (a) the patient’s request is voluntary and well-considered; (b) the patient’s suffering is unbearable and without prospect of relief; (c) the patient is informed about his/her situation and prospects; (d) there is no reasonable alternative solution; (e) another independent physician has consulted the patient; (f) the physician terminated the patient’s life with due medical care and attention [2]. The second independent physician assesses whether the due care criteria (a t/m d) are met and advices the attending physician [3, 4]. In 78% of the consultations a SCEN physician (Support and Consultation on Euthanasia in the Netherlands) is consulted as second independent physician [1]. The SCEN project is a programme of the Royal Dutch Medical Association (RDMA) with the aim to improve the quality of consultation and thereby the quality of EAS [3]. SCEN physicians receive a 3-day training programme in which their tasks and the Dutch Euthanasia Act are discussed [5]. When active as SCEN physician, they are expected to attend intervision three times per year in their SCEN-region. Both training and intervision are considered important to maintain quality of consultation [5].

In recent years, the public debate about EAS has focused on patients with psychiatric disorders, dementia, and older people tired of living as complex cases [6–8]. Nonetheless, these patients can meet the due care criteria. For patients tired of living, an important topic is whether these patients have medically classifiable diseases, which is a requirement to meet the due care criteria. These are currently frequently operationalized as accumulation of age-related health problems in the tired of living debate. In 2016, 4% of the requests was underlain by a psychiatric disorder, 3% by dementia and 6% by no serious physical or psychiatric illness [1]. According to a study among attending physicians, significantly less physicians found EAS conceivable for patients with a psychiatric disease, dementia or those being tired of living compared to patients with cancer or other physical diseases [7].

Yet, these complex cases present in the public debate might not cover all complexities that can arise in EAS practice. Research has been conducted among attending physicians, but SCEN physicians differ from attending physicians with regard to three aspects. First, they are specifically trained in assessing the due care criteria and have the most experience. Therefore, they are the best to provide information on complexities. Second, they have a different relationship with the patient. Whereas an attending physician has a long-term relationship with the patient, the SCEN physician often consults the patient once to assess the due care criteria. Third, they fulfil another role in the process of EAS compared to the attending physician. They assess the criteria based on the law, and thus, unlike attending physicians, they do not have to take their personal boundaries into consideration. It stands to reason that SCEN physicians also experience complexities, although those may be different from the attending physicians. However, what complexities SCEN physicians perceive during EAS consultations, is unknown until now. Therefore, the following research questions will be addressed: What is the frequency of consultations that are perceived difficult by SCEN physicians? What complexities do SCEN physicians perceive during consultation? What characteristics are associated with SCEN physicians perceiving a consultation as difficult?

## Methods

### Study design and data collection

From 2008 onwards, an annual survey of RDMA is held among SCEN physicians in the Netherlands to collect specific data on their most recent consultation. Data from 2015, 2016 and 2017 was included in this cross-sectional study. The response rate was 78.7% (*N* = 546) in 2015, 76.4% (*N* = 542) in 2016 and 72.1% (*N* = 498) in 2017. Only questionnaires with complete data for the dependent variables were analysed. This resulted in analysis of 498 questionnaires filled in by 498 SCEN physicians for 2015 and, in total, 938 questionnaires filled in by 573 SCEN physicians for 2016 and 2017. In 2015, the questionnaire focused on the most difficult consultation instead of the most recent consultation. The data from 2015 were used to explore what characteristics of complex consultations are according to SCEN physicians. The data from 2016 and 2017, about the most recent consultation, were used to assess the frequency of consultations perceived difficult by SCEN physicians and what characteristics are associated with consultations that are perceived difficult by SCEN physicians. As the 2015 data only included difficult consultations, these data could not be included in these analyses of difficult versus non difficult consultations.

### Questionnaires

Questionnaires were developed for the purpose of monitoring SCEN and were used in a previous study of Brinkman-Stoppelenburg et al. (2014) [9]. Both the questionnaire in 2015 and the questionnaires in 2016 and 2017 included questions about the amount of consultations in the last year, the setting in which they were provided, and patient characteristics. Specific questions were posed about the most difficult (2015) or most recent (2016/2017) consultation the SCEN physician conducted. In the questionnaire in 2015 an open-ended question was included on what complexities SCEN physicians had perceived. This open-ended question consisted of five prescribed categories for which the SCEN physicians could indicate what they considered complex (if anything in that category) to ensure that respondents answered extensively. The five prescribed categories were patient characteristics, assessment of due care criteria, patient’s relatives, health professionals and other. Based on these categories, questions were formulated for the questionnaire in 2016 and 2017 on perceived difficulty of consultation, for four aspects: the assessment of the due care criteria, patient characteristics, patient’s relatives, and health professionals. The questions had a 5-pointscale (1 = not difficult at all, 5 = extremely difficult) and if they scored 4 or 5 for at least one of the aspects, the consultation was considered as difficult (dichotomous). Hereafter, we will refer to consultations being perceived as difficult by SCEN physicians, as difficult consultations.

### Analysis

Statistical analyses were performed using SPSS IBM 22. Descriptive statistics were used to describe the frequency of characteristics of (difficult) consultations for the most difficult (2015) and most recent consultations (2016/2017) separately. Characteristics of the 2015 sample and the 2016/2017 sample are presented combined in Table 1 for each year separately.

To assess what complexities SCEN physicians perceived during their most difficult consultation (2015), the answers to the open-ended question on perceived complexities were coded by BDOP and HRWP separately and then compared. Frequency analysis of the codes was done to assess how often specific complexities were encountered.

To assess what characteristics are associated with difficult consultations (2016/2017) we included 24 independent variables from three categories. First, patient characteristics were age, sex, residency, main diagnosis, other diagnoses besides the main diagnosis (5 variables), and important reasons to request EAS (11 variables). Second, characteristics of the attending physician included the profession, works at End of Life clinic, and certainty of the attending physician to perform EAS (hereafter certainty attending physician). Third, we included the number of consultations per year as characteristic of SCEN physicians. Several univariable logistic regression analyses and a multivariable logistic regression analysis with difficult consultations as the dependent variable were conducted to assess what characteristics were significantly associated. Characteristics were included in analyses if they counted at least 5 people in each group. Both linearity and multicollinearity were checked. All characteristics that were statistically significant (*p* < 0.05) in univariable logistic regression analyses were included in the multivariable logistic regression analysis. These analyses were repeated for the separate aspects with a prevalence of complexity of at least 10%, namely patient characteristics and assessment of the due care criteria, to assess if there were any differences between these aspects. No analyses were conducted for the aspects interaction with the patient’s relatives and health professionals because the numbers of cases in which these were considered difficult were very low and analyses would therefore not give a precise estimate of the effect due to low power. Sensitivity analysis, with the use of generalised estimating equations (GEE), were used to check for possible clustering within SCEN physicians.

## Results

### Frequency of difficult consultations and characteristics of patients of whom SCEN physicians judged their request for EAS

The sample of 2016 and 2017 consisted of patients of the most recent consultation of the SCEN physician. In 2016 and 2017, 21.6% of consultations were perceived difficult by SCEN physicians: 203 out of 938 consultations. The assessment of due care criteria was most often perceived as difficult (14.8%), followed by patient characteristics (10.3%). Difficult consultations due to interaction with the patient’s relatives and health professionals were less prevalent, namely 4.4 and 3.1%. Cancer was the most prevalent main diagnosis and makes up for 75% of the not difficult cases, while for the most difficult (from 2015) and difficult cases (from 2016/2017) this was significantly lower, namely 34 and 36%. In 2016 and 2017, 29 cases (14%) concerning patients diagnosed with accumulation of age-related health problems were considered difficult and 28 (4%) as not difficult. The diagnoses accumulation of age-related health problems, dementia and psychiatric disorder were significantly less prevalent in not difficult cases compared to (most) difficult consultations (Table 1)

**Table 1:** Characteristics of patients with EAS request assessed by SCEN physicians, by experienced difficulty and year (absolute numbers and rounded percentages)

	2015 Most difficult consultation (*N* = 498)			2016/2017 Most recent consultation					
				Difficult (*N* = 203)			Not difficult (*N* = 735)		
	N	%	(95% CI)	N	%	(95% CI)	N	%	(95% CI)
Age									
< 70 years	208	45	(40–49)	78	39	(32–46)	260	36	(32–39)
70–79 years	101	22	(18–26)	36	18	(13–24)	240	33	(30–36)
≥ 80 years	157	34	(30–38)	86	43	(36–50)	229	31	(28–35)
Sex (female)	255	53	(48–57)	104	52	(44–58)	339	46	(43–50)
Main diagnosis									
Cancer	162	34	(29–38)	73	36	(30–43)	551	75	(72–78)
Heart failure/CVA	42	9	(6–11)	23	11	(8–16)	35	5	(3–6)
MS/ALS	21	4	(3–6)	7	4	(2–7)	22	3	(2–4)
COPD	17	4	(2–5)	7	4	(2–7)	33	5	(3–6)
Accumulation of age-related health problems	53	11	(8–14)	29	14	(10–20)	28	4	(3–5)
Dementia	44	9	(7–12)	17	8	(5–13)	9	1	(1–2)
Psychiatric disorder	46	10	(7–12)	18	9	(6–13)	3	0	(0–1)
Other	99	21	(17–24)	28	14	(10–19)	53	7	(6–9)

Missing values ranging from 2 to 32

.

### Perceived complexities by SCEN physicians during the most difficult consultation

SCEN physicians described what complexities they perceived during their most difficult consultation in an open-ended question included in 2015. Table 2 consists of the results after coding the answers to the open-ended question which was filled in by 449 of the 498 SCEN physicians. These complexities concerned five aspects. First, patient characteristics was the most prevalent described aspect (79.7%) and most mentioned complexities were: no short-term life-threatening disease (18.8%), difficulties in communication (18.4%), psychiatric disorders (13.5%). Second, in 41.0% of the cases the assessment of due care criteria was perceived difficult and most mentioned complexities included: suffering hard to assess due to the situation of the patient (11.8%), early consultation (8.9%), mental competence hard to assess (6.6%). Third, interaction with the patient’s relatives was mentioned as difficult in 26.3% of the cases and most mentioned perceived complexities were: relatives are not ready for/have problems with the EAS (7.5%), relatives exert pressure on the physician (5.6%), problems within the family that are not directly related to the EAS request (4.6%). Fourth, in 23.4% of the cases interaction with the health professionals was perceived as difficult. These mainly concerned problems with the attending physician: pressure/not understanding doubts or decision SCEN physician (8.9%), doubting/unclear/unprepared attending physician (6.1%), attending physician does not want to perform the EAS (2.4%). Fifth, in 13.4% of the cases the perceived complexities concerned other aspects. Examples are that an earlier SCEN physician judged differently (2.9%) and little experience of SCEN physician with disease (2.6%). Table 3 gives examples of quotes about perceived difficulties.

**Table 2:** Perceived complexities by SCEN physicians during the most difficult consultation, 2015

Most difficult consultation *N* = 449* (%)		
Patient characteristics	79.7	
No short-term life-threatening disease (e.g. accumulation of age-related health problems, chronic diseases, invalidity)		18.8
Communication was difficult (e.g. coma, confused, not clear, aphasic)		18.4
Psychiatric problems		13.5
Ambivalence towards death wish		12.8
Patient was demanding, angry		11.6
Dementia, cognitive decline		10.7
Patient was young		7.8
Psychosocial problems (e.g. tired of living, loneliness)		5.8
Time pressure due to medical state		2.6
Assessment of the due care criteria	41.0	
Suffering hard to assess due to the situation of the patient		11.8
An early consultation		8.9
Mental competence hard to assess		6.6
Unbearable suffering hard to imagine		5.8
Treatment options hard to define		5.6
Suffering hard to asses due to lack of information (from patient/file/physician)		4.4
Hard to assess whether the request was voluntary		1.5
Patient’s relatives	26.3	
Relatives aren’t ready for/have problems with the EAS		7.5
Relatives exert pressure on the physician		5.6
Problems within the family (not related to the EAS e.g. grief, no contact with children, family lives far away)		4.6
Patient wants EAS together with partner		2.2
Family cannot handle the care for the patient		2.2
Patient provides care for children/partner		2.2
Pressure from relatives on patient		0.8
Health professionals	23.4	
Pressure/not understanding doubts or judgement of the SCEN physician		8.9
Doubting/unclear/unprepared attending physician		6.1
Attending physician does not want to perform the EAS		2.4
Attending physician was influenced by the patient and relatives		2.3
Suboptimal care (e.g. too little care, not in place wished for, futile treatment)		2.3
Attending physician already promised to perform the EAS		1.9
Bad contact between attending physician and patient		1.7
(Part of) health professionals are against EAS/agitation among health professionals		1.2
Attending physician expected more counseling from the SCEN physician		1.0
Attending physician seemed to want consultation/EAS earlier than patient		0.6
Other aspects	13.4	
An earlier SCEN physician judged differently		2.9
Little experience of SCEN physician with disease		2.6
Resistance of SCEN physician concerning a specific case		2.6
Situation was moving		1.9
Attending physician was also a SCEN physician		0.8
SCEN physician acted awkward him/herself		0.6
Questionable whether the second physician was independent		0.6
Consultation with other SCEN physician necessary		0.6
Other		1.5

*More than 1 answer possible; 449 of 498 SCEN physicians answered this question

**Table 3:** Examples of perceived complexities described by SCEN physicians during the most difficult consultation, 2015

**Patient characteristics** “A vital man; he still walked and cycled” “A young patient with a medical situation that was hard to describe; the patient was critical towards me and was resistant to have a conversation” “Patient had reoccurring doubts about the euthanasia; at moments she seemed convinced, but later she had doubts”	
**Assessment of the due care criteria** “The patient wanted a judgment if euthanasia was possible beforehand; at the time of consultation, unbearable suffering or a request were not present” “The patient was adequate in his response capacities, especially in the presence of relatives. However, when I talked to him alone, the characteristics of his dementia became very clear” “I found the unbearable suffering hard to assess. He said unbearable suffering, but there was still a lot that he enjoyed to do”	
**Patient’s relatives** “The partner was really sad; his vison was not crystallized; paid a lot of attention to” “They were angry; the euthanasia was already promised. Due to a gastrointestinal bleeding, the situation was further worsened, but they still demanded the euthanasia” “Disagreement between the children about their mother’s wish”	
**Health professionals** “I felt pressured. The attending physician wasn’t open for alternative treatment options and advice from me” “The general practitioner was unclear towards me, the patient and himself” “Really odd consult request. Both the general practitioner and the psychiatrist knew that the patient wouldn’t meet the due care criteria. I felt used”	
**Other aspects** “No experience with a young man taking such a decision” “I notice resistance in myself with these chronic physical problems combined with personality problems. Is this the purpose of the euthanasia law?” “For myself heart-breaking. Given the situation, I supported it completely. I experience less resistance with a 90 year old with cancer [case concerning a young woman]”	

### Characteristics associated with difficult consultations as perceived by SCEN physicians

Table 4 presents descriptive statistics, univariable ORs and multivariable ORs of each characteristic of the most recent consultation in 2016 and 2017. Six characteristics remained statistically significant in multivariable analysis as associations with difficult consultations. Compared to attending physicians who already promised to grant the request, attending physicians who had doubts about or did not want to grant the request had 4.48 higher odds of difficult consultations. Consultations concerning patients between 70 and 79 years old (OR = 0.38) and older than 80 years (OR = 0.53) had a lower likelihood of difficult consultations, compared to patients younger than 70 years. Compared to patients staying at home, patients staying at the hospital had 3.01 higher odds of difficult consultations. Compared to cancer patients, patients with health failure or cerebrovascular accident (CVA) (OR = 4.43), patients with accumulation of age-related health problems, dementia or psychiatric disorder (OR = 6.94) or other diagnoses (OR = 3.42), such as Parkinson’s disease (*N* = 13) and lung fibrosis (*N* = 6), had higher odds of difficult consultations. Besides the main diagnosis, the presence of a psychiatric disorder (OR = 3.91) or psychosocial or existential problems (OR = 2.25) had higher odds of difficult consultations. Physical suffering as an important reason for the EAS request had a lower odds of difficult consultations (OR = 0.62). GEE was conducted as sensitivity analysis. The ORs and CIs of the multivariable logistic regression were comparable to the ORs and CIs in the GEE.

**Table 4:** Characteristics associated with difficult consultations as perceived by SCEN physicians, 2016–2017

	Difficult (*N* = 203)	Not difficult (*N* = 735)	Univariable Odds ratio (95% CI)	Multivariable Odds ratio (95% CI)
	N (%)	N (%)		
**SCEN physician**				
Number of consultations per year				
< 5	11 (5.5)	52 (7.1)	1	¶
5–9	53 (26.5)	190 (25.9)	1.32 (0.64–2.7)	
10–14	52 (26)	212 (28.9)	1.16 (0.57–2.38)	
≥ 15	84 (42)	280 (38.1)	1.42 (0.71–2.84)	
**Attending physician**				
Profession				
General practitioner	151 (81.2)	665 (92.7)	1	1
Medical specialist	17 (9.1)	23 (3.2)	3.26 (1.7–6.24)	1.23 (0.48–3.17)
Elderly care physician	18 (9.7)	29 (4)	2.73 (1.48–5.05)	0.73 (0.31–1.69)
Works at End of life clinic	46 (22.8)	49 (6.7)	4.13 (2.66–6.4)	1.44 (0.71–2.94)
Certainty of decision before consultation				
Already promised to grant the request	29 (14.3)	186 (25.4)	1	1
Decided to grant the request	78 (38.4)	353 (48.2)	1.42 (0.89–2.25)	0.76 (0.43–1.33)
Probably wants to grant the request	75 (36.9)	177 (24.1)	2.72 (1.69–4.37)	1.58 (0.88–2.82)
Doubts about/doesn’t want to grant the request	21 (10.3)	17 (2.3)	7.92 (3.74–16.77)	4.48 (1.73–11.58)
**Patient**				
Age				
< 70 years	78 (39)	260 (35.6)	1	1
70–79 years	36 (18)	240 (32.9)	0.5 (0.33–0.77)	0.38 (0.21–0.68)
≥ 80 years	86 (43)	230 (31.5)	1.25 (0.88–1.78)	0.53 (0.31–0.93)
Sex (female)	104 (51.7)	339 (46.2)	1.25 (0.91–1.71)	¶
Residency				
Home	136 (67.7)	615 (84)	1	1
Care/nursing home	33 (16.4)	57 (7.8)	2.62 (1.64–4.18)	1.31 (0.7–2.47)
Hospital	17 (8.5)	20 (2.7)	3.84 (1.96–7.53)	3.01 (1.15–7.85)
Hospice	15 (7.5)	40 (5.5)	1.7 (0.91–3.16)	2.01 (0.9–4.49)
Main diagnosis				
Cancer	73 (36.1)	551 (75.1)	1	1
Heart failure/CVA	23 (11.4)	35 (4.8)	4.96 (2.78–8.86)	4.43 (2.08–9.42)
MS/ALS	7 (3.5)	22 (3)	2.4 (0.99–5.82)	0.85 (0.25–2.87)
COPD	7 (3.5)	33 (4.5)	1.6 (0.68–3.75)	0.7 (0.2–2.46)
Accumulation of age-related health problems/dementia/psychiatric disorder	64 (31.7)	40 (5.4)	12.08 (7.59–19.21)	6.94 (3.58–13.46)
Other	28 (13.9)	53 (7.2)	3.99 (2.37–6.7)	3.42 (1.77–6.6)
Besides main diagnosis also^a^				
Physical illness	94 (50.5)	299 (49.9)	1.03 (0.74–1.43)	¶
Psychiatric disorder	29 (15.6)	53 (8.8)	5.17 (2.28–11.71)	3.91 (1.41–10.83)
Accumulation of age-related health problems	29 (15.6)	53 (8.8)	1.9 (1.17–3.1)	1.58 (0.77–3.24)
Dementia	6 (3.2)	9 (1.5)	2.19 (0.77–6.22)	¶
Psychosocial or existential problems	34 (18.3)	31 (5.2)	4.1 (2.44–6.88)	2.25 (1.13–4.5)
Reasons to request EAS				
Loss of dignity	90 (44.3)	364 (49.6)	0.81 (0.59–1.11)	¶
Overall weakness	86 (42.4)	457 (62.3)	0.45 (0.33–0.61)	0.78 (0.51–1.2)
Being tired with life	17 (8.4)	20 (2.7)	3.26 (1.68–6.35)	0.95 (0.37–2.43)
Pointless suffering	45 (22.2)	156 (21.3)	1.06 (0.73–1.54)	¶
Knowing that suffering will not get better	93 (45.8)	382 (52)	0.78 (0.57–1.06)	¶
Fear of suffocation	15 (7.4)	89 (12.1)	0.58 (0.33–1.02)	0.73 (0.32–1.67)
Invalidity	42 (20.7)	115 (15.7)	1.4 (0.95–2.08)	¶
Depression^b^	12 (5.9)	1 (0.1)	–	–
Dependency	92 (45.3)	353 (48.1)	0.9 (0.66–1.22)	¶
Not wanting to be a burden for family	22 (10.8)	32 (4.4)	2.67 (1.51–4.7)	1.32 (0.58–2.99)
Physical suffering^c^	72 (35.5)	405 (55.2)	0.45 (0.32–0.62)	0.62 (0.4–0.96)

*N* number, *CI* confidence interval; ¶, not significant in univariable analysis, thus not included in multivariable analysis; ^a^Missing values = 153; ^b^Not included in analysis due to small sample size; ^c^Includes pain, vomiting and dyspnea; Missing values ranging from 1 to 35

12 out of 13 cases that concerned patients that requested EAS because of depression were difficult cases. However, with only one case for which the consultation was not difficult, depression was not included in analyses.

### Characteristics associated with difficult consultations as perceived by SCEN physicians for the assessment of due care criteria and patient characteristics

For the two aspects that were most frequently considered difficult, namely the assessment of due care criteria and patient characteristics, we performed separate multivariable analyses (Table 5). Four characteristics of consultations remained significantly associated with perceived difficulty of the assessment of due care criteria are. Compared to attending physicians who already promised to grant the request, attending physicians who had doubts about or did not want to grant the request had 4.69 higher odds of difficult assessment of due care criteria. Compared to patients aged younger than 70 years, patients aged between 70 and 79 years (OR = 0.37) and older than 80 years (OR = 0.41) had a lower odds of difficult assessment of due care criteria. Patients with the main diagnoses heart failure or cerebrovascular accident (CVA) (OR = 3.45), accumulation of age-related health problems, dementia or psychiatric disorder (OR = 13.52) or other diagnoses (OR = 5.05) had a higher odds of difficult assessment of due care criteria, compared to patients with cancer. Patients with psychosocial or existential problems besides their main diagnosis had 2.25 higher odds of difficult assessment of due care criteria, compared to patient without these problems.

**Table 5:** Characteristics associated with consultations perceived as difficult by SCEN physicians for the assessment of due care criteria and patient characteristics, 2016–2017

	Multivariable odds ratio (95% CI)	
	The assessment of due care criteria	Patient characteristics
Difficult (score ≥ 4)	139 (14.8)^a^	97 (10.3)^a^
**SCEN physician**		
Number of consultations per year		
< 5	¶	¶
5–9		
10–14		
≥ 15		
**Attending physician**		
Profession		
General practitioner	1	1
Medical specialist	0.93 (0.32–2.73)	3.02 (1.09–8.35)
Elderly care physician	0.62 (0.24–1.61)	0.99 (0.37–2.68)
Works at End of life clinic	1.82 (0.85–3.89)	0.64 (0.25–1.63)
Certainty of decision before consultation		
Already promised to grant the request	1	1
Decided to grant the request	0.72 (0.36–1.44)	0.74 (0.35–1.55)
Probably wants to grant the request	1.93 (0.97–3.83)	1.27 (0.6–2.7)
Doubts about/doesn’t want to grant the request	4.69 (1.71–12.88)	3.52 (1.22–10.18)
**Patient**		
Age		
< 70 years	1	1
70–79 years	0.37 (0.18–0.75)	0.41 (0.18–0.92)
≥ 80 years	0.41 (0.21–0.79)	1 (0.52–1.89)
Sex (female)	¶	¶
Residency		
Home	1	1
Care/nursing home	1.52 (0.76–3.05)	1.72 (0.8–3.69)
Hospital	1.45 (0.48–4.41)	5.08 (1.8–14.31)
Hospice	2 (0.76–5.24)	3.27 (1.32–8.1)
Main diagnosis		
Cancer	1	1
Heart failure/CVA	3.54 (1.44–8.66)	2.17 (0.87–5.39)
MS/ALS	0.79 (0.16–3.89)	0.94 (0.2–4.45)
COPD	1.18 (0.29–4.75)	0.66 (0.15–2.89)
Accumulation of age-related health problems/dementia/psychiatric disorder	13.52 (6.4–28.56)	2.91 (1.31–6.43)
Other	5.05 (2.42–10.53)	1.35 (0.56–3.27)
Besides main diagnosis also^b^		
Physical illness	¶	¶
Psychiatric disorder	¶	8.24 (3.11–21.86)
Accumulation of age-related health problems	1.96 (0.87–4.46)	¶
Dementia	¶	¶
Psychosocial or existential problems	2.25 (1.07–4.71)	2.24 (1.07–4.72)
Reasons to request EAS		
Loss of dignity	¶	¶
Overall weakness	0.93 (0.57–1.54)	1.22 (0.69–2.17)
Being tired with life	0.94 (0.34–2.57)	1.47 (0.52–4.18)
Pointless suffering	¶	¶
Knowing that suffering will not get better	¶	¶
Fear of suffocation	0.62 (0.21–1.8)	¶
Invalidity	¶	¶
Depression	–	–
Dependency	¶	¶
Not wanting to be a burden for family	1.67 (0.7–4)	1.3 (0.49–3.5)
Physical suffering^c^	0.69 (0.41–1.15)	0.87 (0.49–1.52)

*N* number, *CI* confidence interval; ¶, not significant in univariable analysis, thus not included in multivariable analysis; ^a^Number (percentage); ^b^Missing values = 153; ^c^Includes pain, vomiting and dyspnea; Missing values ranging from 1 to 35

Seven characteristics remained significantly associated with characteristics of patients that contribute to a consultation being perceived difficult. Compared to the attending physicians being a general practitioner, the attending physician being a medical specialist had a 3.02 higher odds of characteristics of patients that contribute to a consultation being perceived difficult. Compared to attending physicians who already promised to grant the request, attending physicians who had doubts about or did not want to grant the request had 3.52 higher odds of characteristics of patients that contribute to a consultation being perceived difficult. Patients aged between 70 and 79 years had a lower odds of characteristics of patients that contribute to a consultation being perceived difficult (OR = 0.41), compared to patients younger than 70. Compared to patients staying at home, patients staying in a hospital (OR = 5.08) or hospice (OR = 3.27) had higher odds of characteristics of patients that contribute to a consultation being perceived difficult. Consultations concerning patients with accumulation of age-related health problems, dementia or a psychiatric disorder as main diagnosis had higher odds of characteristics of patients that contribute to a consultation being perceived difficult (OR = 2.91), compared to patients with cancer. The presence of a psychiatric disorder (OR = 8.24) or psychosocial or existential problems (OR = 2.24) besides the main diagnosis had higher odds of characteristics of patients that contribute to a consultation being perceived difficult.

## Discussion

We found that one out of five EAS consultations is perceived difficult by SCEN physicians. Perceived complexities are mostly related to the patient characteristics and the assessment of due care criteria. Characteristics that are associated with a higher likelihood of difficult consultations are the certainty of the attending physician on whether or not to perform the EAS before the consultation with the SCEN physician (not already promised to grant the request), patient’s residence (not home), main diagnosis of the patient (not cancer), and the presence of a psychiatric disorder or psychosocial or existential problems besides the main diagnosis. Characteristics that are associated with a lower likelihood of difficult consultation are high patient’s age and physical suffering as important reason to request EAS.

### Frequency of consultations that are perceived difficult

Over one out of five consultations is perceived difficult by SCEN physicians. If we multiply with all consultations in 2016 [10], approximately 1750 of all consultations that were performed by SCEN physicians in 2016 are possibly perceived difficult. 1750 is substantial and it would be good if this would be subject of discussion in intervision. This difficulty is not necessarily problematic. EAS is an inherently sensitive subject that needs careful consideration. With regard to SCEN physicians performing their work in the review process, it is especially important that they are able to assess the due care criteria. The fact that SCEN physicians perceive difficulties does not necessarily mean that there are problems with the assessment of due care criteria. SCEN physicians received training in assessing the due care criteria and thus have the most experience to deal with complexities.

### Characteristics associated with difficult consultations

In recent years, the public debate mainly focuses on patients with dementia, psychiatric disorder, or accumulation of age-related health problems, as difficult cases [6–8]. Our findings confirm that these cases are often perceived difficult by SCEN physicians, as we found a strong association between these diagnoses and perceived difficulty, and as they were mentioned by SCEN physicians as complexities. This is in agreement with results obtained by Bolt et al. (2015) in a study among attending physicians [7]. One explanation for these cases being perceived difficult can be related to the difficulty of assessing whether suffering is unbearable in these patient groups, where psychosocial suffering is mainly present. Besides, from previous research we know that physicians are less likely to judge suffering unbearable in psychosocial suffering, present in for example early stage dementia, compared to physical suffering [7, 11]. Other explanations may be the uncertainty about the availability of alternative treatment options in patients with psychiatric disorders and about whether the request was voluntary and well-considered in patients with dementia [7, 12]. These explanations were also mentioned separately as complexities by SCEN physicians (Table 3).

Also, consultations concerning patients with a psychiatric disorder besides their main diagnosis are perceived difficult more than patients without a psychiatric disorder. Yet, SCEN physicians do not find it more difficult to judge the due care criteria for this specific patient group, but do perceive more difficulty in contact with the patient. An explanation may be that with the assessment of the due care criteria the main diagnosis suffices, and thus the psychiatric disorder on top of that does not necessarily complicate this assessment.

Not only the ‘complex’ cases present in the public debate, patients with dementia, psychiatric disorder, or accumulation of age-related health problems, are perceived as difficult. Patients with the diagnosis heart failure or CVA are more often perceived difficult by SCEN physicians compared to cancer patients. The unpredictable course of the heart failure or CVA disease trajectory, compared to predictability of cancer trajectory, might account for some of the difficulty in characterizing the process of dying [13–15]. This unpredictability may complicate the assessment of the criterion unbearable suffering without prospect of relief. Also, consultations concerning patients with other diagnoses (e.g. Parkinson’s disease, lung fibrosis) are perceived difficult more often than cancer patients. Explanations may be that these concern diagnoses that SCEN physicians have not seen a lot or are not familiar with, and are therefore harder to assess, which is also mentioned by SCEN physicians (Table 3). Another explanation may be that these other diagnoses are in themselves difficult.

Not only the patient’s diagnosis is associated with perceived difficulty of consultations. First, if the patient has a high age (≥ 70 years), the likelihood of difficult consultations as perceived by SCEN physicians is lower, compared to patients with a lower age (< 70 years). Older people have a reduced life expectancy compared to younger people and therefore an EAS request from younger people could be more confronting for SCEN physicians. Second, patients staying in the hospital have a higher likelihood of difficult consultations than patients staying at home. SCEN physicians experience difficulties with the patient, and not in assessing the due care criteria. Hospitalization at the end of life often takes place because of an acute medical situation [16], and this may cause difficulties in interaction with the patient, such as stress. Third, the uncertainty of the attending physician to grant the request is associated with perceived difficulty for the SCEN physician and was mentioned by SCEN physicians as complexity in more than one way. This may be explained by difficulties that arise from different opinions of the attending physician and SCEN physician or uncertainty about this decision because the EAS request or the situation in itself is complex.

### Strengths and limitations

A major strength of this study is the high number of cases and response rate. Another strength is the inclusion of both an open-ended question on descriptions of perceived complexities during the most difficult consultation, and analyses to assess which characteristics are associated with difficult consultations. Both the description of perceived complexities as the analyses of characteristics associated with difficult consultations have similar results which adds to the reliability of conclusions made in this study. A limitation is the small numbers of patients with some specific characteristics which resulted in wide confidence intervals. Therefore, these results should be interpreted with caution. Recall bias could be another limitation and results in SCEN physicians not remembering the request well. On the other hand, complex cases are probably remembered better than not complex cases. Another limitation is the small amount of characteristics of SCEN physicians in analyses. Further research should take these into account.

## Conclusions

Complexities perceived by SCEN physicians in EAS consultations are not limited to the ‘complex’ cases present in the current public debate about EAS, e.g. patients with psychiatric disorders, dementia, and tired of living. Difficult consultations perceived by SCEN physicians are not rare, as they occur in one out of five consultations. This difficulty is not necessarily problematic, as EAS is a sensitive subject. Yet, attention in the SCEN program is needed for these complex consultations. Training and intervision is already implemented in the SCEN program. Attention for these complexities in intervision could indicate if there is a need among SCEN physicians to enhance knowledge and skills in training and to receive specific support in intervision on these complexities.

## Data Availability

The datasets used and/or analysed during the current study are available from the corresponding author on reasonable request.

## Abbreviations

CI: Confidence interval
EAS: Euthanasia and physician-assisted suicide
OR: Odds ratio
RDMA: Royal dutch medical association
